# The impostor phenomenon in the eye of knowledgeable others: the association of the impostor phenomenon with the judge’s accuracy

**DOI:** 10.3389/fpsyg.2023.1290686

**Published:** 2023-12-22

**Authors:** Fabio Ibrahim, Erik Brill, Tobias Meyberg, Philipp Yorck Herzberg

**Affiliations:** ^1^Department of Personality Psychology and Psychological Assessment, Helmut-Schmidt-University, Hamburg, Germany; ^2^European University of Applied Sciences for Distance Learning Hamburg, Hamburg, Germany

**Keywords:** impostor phenomenon, profile agreement, impostor-profile, informant assessment, self-other agreement, distinct agreement

## Abstract

This study examines whether a heightened impostor expression is associated with a judge’s assessment. The sample comprised *n* = 155 triads (target, mother, and a friend). Results indicated a slightly higher profile agreement between the target and mother (*r*_raw_ = 0.47; *r*_distinct_ = 0.33) than a friend (*r*_raw_ = 0.41; *r*_distinct_ = 0.23). The profile agreement was inversely correlated with the IPP total score, Competence Doubt, Alienation, and Other-Self Divergence (*r* = ≤ −0.29, *p* < 0.001), indicating reduced accuracy among judges when confronted with a heightened impostor expression. However, these relationships disappear once controlling for stereotype effects. Overall, this study reveals a negative association between the impostor expression and the other-self agreement, supporting the biasing self-presentation of impostors in the eye of others.

## 1 Introduction

The Impostor Phenomenon (IP), which characterizes high-achievers who underestimate their abilities, arises from a deficiency in internalizing success despite objective achievement indicators (Clance, 1985). This maladaptive attribution diminishes self-esteem (Ibrahim et al., 2022a) and leads to a heightened fear of failure (Noskeau et al., 2021). Those affected fear being exposed (Vergauwe et al., 2015), show elevated levels of social anxiety (Yaffe, 2021), and a perfectionistic self-presentation (Ferrari and Thompson, 2006). Thus, a crucial facet of the IP revolves around concealing self-doubts and cultivating a perfectionistic image in the eyes of others. Following this reasoning, individuals demonstrating heightened impostorism will likely demonstrate a biased self-presentation, leading to a potentially inaccurate external assessment. Although existing research highlights a reduced self-assessed authenticity experience among those with impostor tendencies (Ibrahim et al., 2021), a gap persists in the literature regarding the influence of the IP on the agreement between other and self-assessments, as well as on the overall impression formed by knowledgeable others. Consequently, examining the yet unknown association between the expression of the IP and self-other agreement emerges as a novel aspect in investigating the construct’s implications for interpersonal dynamics and external perceptions. Examining the relationship between self-other agreement and impostorism could serve as an additional criterion to support the overall validity of the construct. To our best knowledge, no prior study examined the IP by incorporating the informant perspective, necessitating the current study’s focus on addressing this still open research question.

## 2 The impostor phenomenon

The IP, initially defined by Clance (1985), encompasses feelings of incompetence, intellectual fraudulence, fear of failure, pre- or procrastination tendencies, and overly ambitious self-expectations. The paradox of feeling inadequate despite notable achievements can be explained by the external-unstable success and internal-stable failure attribution, which have been supported psychometrically (Brauer and Wolf, 2016) and experimentally (Ibrahim et al., 2022c). According to Clance and Imes (1978), the original construct definition included the assumption that the IP affects women, in particular. The findings on the relationship between IP and gender are not yet conclusive. Some studies have found that women (Jöstl et al., 2012) and men (Kolligian and Sternberg, 1991) are more likely to be affected. However, the meta-analysis by Bravata et al. (2020) showed that most studies found no difference between males and females. However, previous studies on the relationship between gender and the IPP have shown that gender differences are particularly evident at the subscale level (Ibrahim et al., 2021) and that different forms of the IP are, therefore, more typical for the genders. In addition to gender, Clance (1985) formulated further explanatory approaches for the emergence of the IP. According to Clance (1985), one etiological factor is a divergence between the person affected and the family’s educational level. Accordingly, those affected feel that they do not belong in the higher socio-economic milieu and feel like a fraud. Another predictor regarding the IP is age, which reduces the impostor expression (Thompson et al., 1998). Higher-aged people are less affected by the IP, possibly as the general experience of authenticity increases over a person’s lifetime (Seto and Schlegel, 2018), as impostors show less self-disclosure (Klinkhammer and Saul-Soprun, 2009) and keep their feelings to themselves (Clance, 1985). Accordingly, the IP aligns with impression management (Parkman, 2016) and maladaptive perfectionism (Brennan-Wydra et al., 2021). Also, the negative correlation of the IP with the HEXACO Honesty-Humility scale and the positive correlation with the scales Situational Variability and Attention to Social Comparison (Ibrahim et al., 2021) underscores the impostor’s attention and value for the external image. Accordingly, Kolligian and Sternberg (1991) called the construct *perceived fraudulence*, emphasizing the divergence between the self- and the perceived other-perception. From a judge’s perspective, the impostor expression is a supposedly hardly assessable trait, as internal cognitions such as anxiety are less observable (Vazire and Carlson, 2010). Accordingly, to increase the judge’s other-assessment validity, multiple and highly knowledgeable raters are necessary (Kenrick and Funder, 1988). In addition, the study of Garwick et al. (2011) indicated, that the relationship quality with the mother was negatively associated with the IP expression. Therefore, we chose the target’s mother and a friend as informants to increase the number of knowledgeable judges, contributing to a more robust external assessment of the IP.

## 3 The present study

With this study, we aim to examine the association of the impostor expression with the other-raters’ accuracy. For this purpose, the Impostor-Profile 30 (Ibrahim et al., 2021) was assessed in a self- and other-assessment version. The multidimensional IPP enabled the measurement of the overall IP expression and the nuanced investigation of the subscales. We used the profile agreement approach, according to Furr (2008), by distinguishing between *normative agreement* (correlation of norm profiles), *overall agreement* (correlation of target and informant profiles), and *distinctive agreement* (correlation of norm-adjusted IPP self- and other-assessments). As a prerequisite, we also evaluated the psychometric properties of the informant IPP30 questionnaire. Furthermore, we explored the correlation between the IP and gender, as this association has not been conclusively answered yet.

*H1*: The other-assessment version of the Impostor-Profile demonstrates structural, content, and empirical comparability with the self-assessment version.

*H2*: The age is negatively associated with the impostor expression.

*H3*: An educational difference between the mother and the child, with the child having a higher degree, is positively associated with the impostor expression.

*H4*: Other-Self Divergence und Alienation are negatively associated with the self-other agreement.

*H5*: The impostor expression is negatively associated with the self-other agreement.

## 4 Methods

### 4.1 Sample

The sample was generated as part of two master’s theses, resulting in two survey periods from May to June 2021 and November 2021 to February 2022, with all participants granting informed consent. The online survey was conducted using Unipark (EFS Survey Version 21.1). A target-created pseudonym connected the data sets, and only complete triads were considered for analysis. After the outliers exclusion, the sample comprised in total *n* = 384 self-ratings (69% females; *M*_age_ = 35.68, *SD* = 15.86 years) and *n* = 310 other-ratings (75% females; *M*_age_ = 16.62, *SD* = 16.56 years; 50% mothers and 50% friends). Overall, the sample consisted of *n* = 155 complete triads.

The targets were predominantly individuals with a school leaving examination (44%) or a bachelor’s degree (34%) as their highest educational attainment, with the majority being university students (57%). Similar characteristics were observed in the demographic profile of friends, with the largest proportion holding a school leaving examination (41%) or a bachelor’s degree (21%) as their highest educational qualification, and the majority identified themselves as students (47%). Among the mothers, the prevailing educational backgrounds included vocational education (32%) and master’s degrees or higher (24%; see Supplementary Table A.1). The university bulletin (students could receive a subject hour for participating), LinkedIn, Xing, and private contacts (Whatsapp) were used for sample acquisition.

An *a priori* power analysis determined a sample size of *n* = 85, offering 80% statistical power and an alpha error of 0.05 based on an anticipated effect size of *r* = 0.30. To estimate the effect size, we used Connelly and Ones’ (2010) meta-analytic results of self-other correlations of neuroticism ( $\bar{r}$ = 0.33). The data is online accessible: https://osf.io/yh5g7.

### 4.2 The Impostor-Profile 30

The Impostor-Profile 30 (IPP; Ibrahim et al., 2021) comprises 30 items (e.g., “Despite former successes, I have a strong fear of failure.”) and measures the impostor expression (IPP total score) and the facets (six subscales) with a 10-point Likert scale ranging from 1 (*Not like me at all*) to 10 (*Very much like me*). The informant version of the IPP consists of the same items with minor adaptations (e.g.: “*Despite former successes, my child has a strong fear of failure*”). In prior investigations of the IPP structure, various models, including unidimensional, multidimensional, second-order, and bifactor models, were compared, revealing that the bifactor model demonstrated the best fit (Ibrahim et al., 2021, 2022b). Furthermore, the bifactor model offers a psychometric benefit by allowing the calculation of measurement invariance at the group factor level (Reise et al., 2010).

### 4.3 Data analysis

We have included only complete triads. Initially, an outlier analysis was performed using Mahalanobi’s distance measure. Four data sets with a highly significant d-squared value (*p* < 0.001) were excluded. In the first step, we calculated the measurement invariance between the self- and other-ratings, which is considered a prerequisite for reasonable comparison, according to Mõttus et al. (2020). Given that previous studies on the IPP have consistently demonstrated the bifactorial structure as best fitting and recognizing the models´ option to compare invariance across group factors (Reise et al., 2010), we used the same bifactor model for the other-assessment version of the IPP for examining the measurement invariance between the IPP’s self- and other-version. In addition, the bifactorial model allowed the contrasting investigation of the subscales’ external assessability to determine more and less observable construct elements of the IP and validate subscales that measure inauthenticity and other-self divergence. We calculated the model fit using the Robust Likelihood estimator with the R software (R Core Team, 2017) and the package lavaan (Rosseel et al., 2017). The measurement invariance of the other- and self-reports was examined in three sequential stages: (a) configural measurement invariance (same item-factor loadings between self- and other-ratings), (b) metric measurement invariance (additionally, factor loadings are constrained equally), (c) scalar measurement invariance (additionally, indicator intercepts are invariant). Following Chen’s (2007) established thresholds, metric invariance was rejected when surpassing ΔCFI ≥ 0.010 and ΔRMSEA ≥ 0.015 (or ΔSRMR ≥ 0.030), while scalar measurement invariance was rejected by exceeding ΔCFI ≥ 0.010 and ΔRMSEA ≥ 0.015 (or ΔSRMR ≥ 0.010).

To examine profile agreement, we used Furr’s (2008) approach by calculating three indicators of agreement: (a) *normative agreement* (agreement between targets’ and judges’ average expressions) as an indicator of the stereotype agreement between target and informant; (b) *overall agreement* (profile correlation between target and mother or friend) as an indicator of self-other agreement; (c) *distinctive agreement* (norm-adjusted profile correlation between target and mother or friend) as an indicator of non-standard agreement between target and informant ratings. To evaluate the profile agreement, we examined overall and distinctive agreement. As an indicator of reliability and to examine different perspectives between the judges, we also calculated the normative, overall, and distinctive inter-rater agreement between the external assessments of mothers and friends.

## 5 Results

### 5.1 Preliminary analysis

The descriptive statistics of the self-other ratings are shown in Table 1. Reliability assessment demonstrated that the IPP had an acceptable to very good reliability in the self-rating version (ɑ = 0.66–0.91), except for the Need for Sympathy scale, which revealed lower reliability (ɑ = 0.55). However, this could be a result of the small number of items, consistent with prior research findings (Ibrahim et al., 2021). The reliability of the other-rating version of the IPP were also acceptable to very good (ɑ = 0.63–0.93), although again, the Need for Sympathy scale had low reliability (ɑ = 0.58).

**Table 1:** Descriptive statistics of the self-other impostor-profile versions and differences between the target and informant ratings.

	Self-reports			Other-report (mother)			Other-report (friend)				
	*M*	*SD*	*α*	*M*	*SD*	*α*	*M*	*SD*	*α*	*d_self-mother_*	*d*_self-friend_
IPP-total score	5.40	1.28	0.90	4.57	1.31	0.90	4.68	1.24	0.89	0.64	0.58
Competence-doubt	5.10	1.97	0.91	4.15	1.96	0.90	4.55	2.03	0.93	0.48	0.28
Working style	6.15	2.23	0.91	5.18	2.27	0.86	4.93	2.25	0.90	0.43	0.54
Alienation	3.52	2.19	0.89	2.55	1.93	0.85	2.96	2.17	0.89	0.47	0.26
Other-self divergence	4.62	2.19	0.85	2.98	1.68	0.75	3.33	1.52	0.72	0.89	0.73
Ambition	5.83	1.97	0.66	6.4	2.29	0.78	5.83	1.84	0.63	0.27	0.00
Need for sympathy	7.51	1.57	0.55	7.2	1.71	0.58	6.99	1.77	0.72	0.18	0.31

*N* = 465; Cohen’s *d* describes the self-other differences between the target and the mother or the friend; range: 1–10.

**Figure 1:**
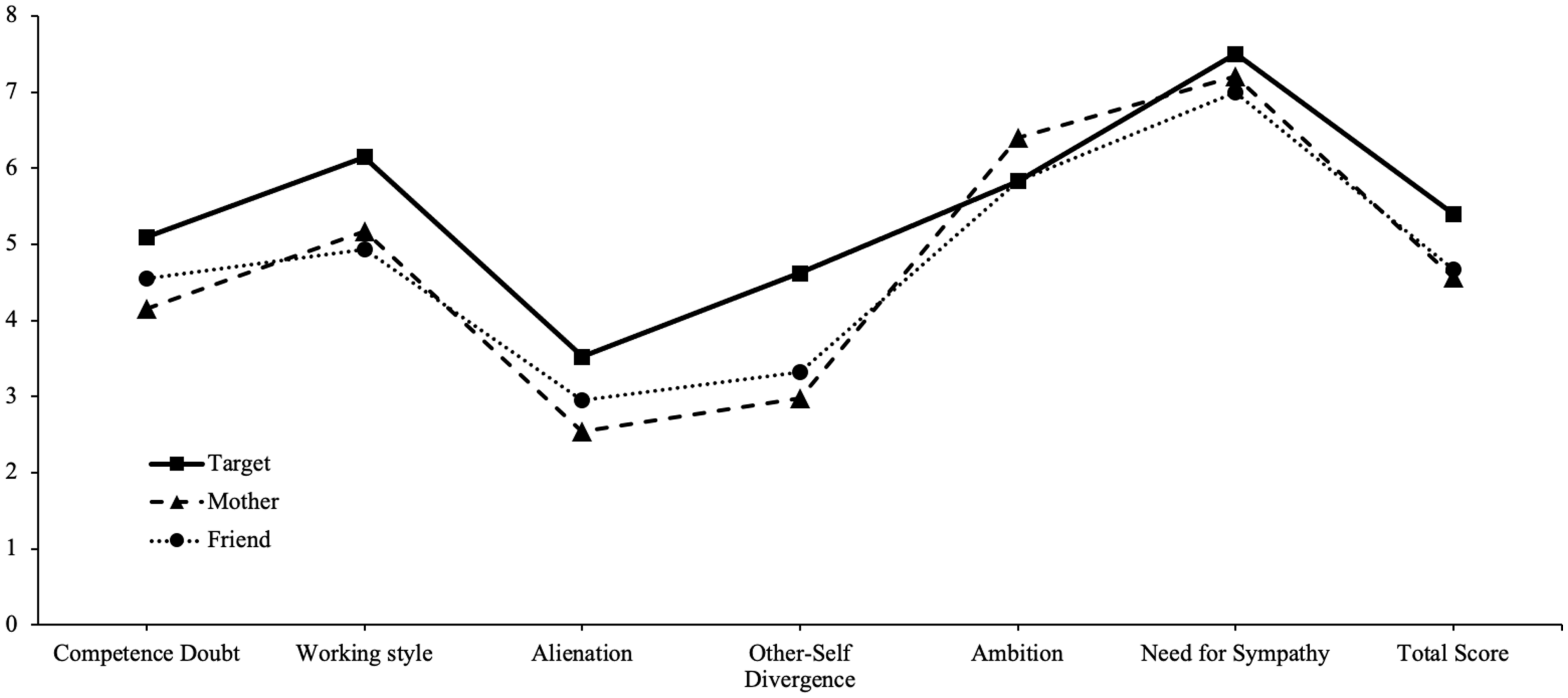
Comparison of the normative impostor-profiles between the target and the informant ratings of the mother and a friend.

Overall, the mean differences between self- and other-ratings were moderate (*ds* ≥ 0.26), with the Need for Sympathy scale showing a lower difference (Table 1). The most substantial differences between the other-self ratings were observed for the IPP subscale Other-Self Divergence (*d*_Mother_ = 0.89; *d*_Friend_ = 0.73) and the IPP total score (*d*_Mother_ = 0.64; *d*_Friend_ = 0.58; Figure 1). The largest differences between the informants consisted between the subscales Alienation (Δ*d* = 0.21) and Ambition (Δ*d* = 0.26).

### 5.2 Measurement invariance and demographic relations

The analysis of measurement invariance (MI) supported scalar measurement invariance between the self- and other-reports (Supplementary Table A.2). This validates the comparability of the IPP’s self- and other versions both in terms of meaning and psychometric properties (H1).

The correlation analyses showed that age, in particular, was negatively related to the IP total score (*r* = −0.25, 95% CI [−0.155, −0.342], *p* < 0.001; H2), with the Ambition subscale showing the largest negative correlation (*r* = −0.31, 95% CI [−0.208, −0.411], *p* < 0.001). Educational level was unrelated to the impostor expression (*r* = −0.07, 95% CI [0.027, −0.173], *p* = 0.152). Similarly, a high educational difference between the mother and informant did not show a relationship with the child’s IP expression (*r* = −0.14, 95% CI [0.017, −0.292], *p* = 0.080; H3).

Recognizing the large number of mothers within the sample, yielding a potential bias in the age-gender distribution, we controlled for age when investigating gender effects. Interestingly, no significant gender differences were observed regarding the IPP total score (*r* = 0.01, 95% CI[0.089, −0.109], *p* = 0.895), although some gender differences emerged at the subscale level. Here, women exhibited higher scores in Competence Doubt (*r* = 0.13, 95% CI[0.227, 0.034], *p* = 0.009) and Alienation (*r* = −0.11, 95% CI [−0.008, −0.213], *p* = 0.035). Conversely, men were higher in Working Style (*r* = −. 17, 95% CI [−0.071, −0.264], *p* < 0.001) and Ambition (*r* = −0.12, 95% CI [−0.025, −0.208], *p* = 0.020), aligning with prior findings (Ibrahim et al., 2021).

### 5.3 Profile agreement

The normative agreement, describing the correlation of norm profiles between the target and the judge, was substantial (*r* ≥ 0.87, *p* < 0.001), with no differences between mothers and friends (Δ*r* = 0.01; Table 2). Comparatively, the overall agreement between the target and mother (*r* = 0.47, *p* < 0.001) was slightly higher than between the target and friend (*r* = 0.41, *p* < 0.001), further increased by aggregating the two other-ratings (*r* = 0.53, *p* < 0.001). Furthermore, the distinctive agreement indicating unique target characteristics, was also higher between the target and mother (*r* = 0.29, *p* < 0.001) than between the target and friend (*r* = 0.23, *p* = 0.002) and further increased by combining the two other-ratings (*r* = 0.33, *p* < 0.001).

**Table 2:** Profile agreement overview between target and informants.

	Mother	Friend	Informant total	Inter-rater
Normative	0.87 [0.94, 0.74]	0.89 [0.95, 0.78]	0.88 [0.94, 0.76]	0.98 [0.95–0.99]
Overall	0.47 [0.41, 0.52]	0.41 [0.36, 0.46]	0.53 [0.47, 0.58]	0.45 [0.39–0.52]
Distinctive	0.29 [0.23, 0.34]	0.23 [0.17, 0.28]	0.33 [0.27, 0.38]	0.20 [0.13–0.26]

*N* = 465; 95% Bootstrapped CI (k = 5,000 samples); Normative, correlation of groups norms; Overall, correlation between self- and informant rated profiles; Distinctive, correlation between self- and informant rated profile corrected for stereotype effects; Inter-rater as profile agreement between the external assessments of the mother and the friend.

Further, we explored the association of gender with the IP, as this debate is still prevalent in IP research. While mothers exhibited a higher profile agreement, the difference to the friends other-rating was not substantial (t_overall_ (308) = 1.433, *p* = 0.153; t_distinctive_ (308) = 1.464, *p* = 0.166). We calculated the inter-rater agreement (profile agreement between external assessments of mother and friend) to determine the reliability. The normative inter-rater agreement was very high (*r* = 0.98). The overall inter-rater agreement lay between the overall profile agreement of mother and friend (*r* = 0.45). The distinctive inter-rater profile agreement was lower than the target-informant distinctive agreements (r = 0.20; Table 2).

To assess potential links between individual facets of the IP and self-other agreement (SOA), we correlated both overall and distinctive agreement with the IPP subscales. The results between the target and the mother indicated that the subscales Alienation (*r*_overall_ = −0.39, *p* < 0.001) and Other-Self Divergence (*r*_overall_ = −0.40, *p* < 0.001) negatively relate to the profile agreement. Similar but weaker relations were apparent between the target and the friend (H4). However, those disappeared when controlling for stereotype effects (distinctive agreement; Table 3). Furthermore, the IPP total score was also negatively related to the profile agreement of the mother (*r*_overall_ = −0.31, *p* < 0.001; H5), whereas the friends’ informant ratings were not associated to the IPP total score (*r_overall_* = −0.12, *p* = 0.133). Again, the effect disappeared when controlling for stereotype effects (*r*_distinctive_ = −0.08, *p* = 0.313).

**Table 3:** Correlation of the targets impostor-profile with profile agreement coefficients.

	Mother		Friend		Informant total	
	Overall	Distinctive	Overall	Distinctive	Overall	Distinctive
s	−0.31^***^	−0.08	−0.12	0.12	−0.29^***^	0.01
Competence-doubt	−0.34^***^	−0.08	−0.08	0.18^*^	−0.29^***^	0.06
Working style	<−0.01	−0.03	0.07	0.05	0.02	0.01
Alienation	−0.39^***^	−0.08	−0.22^**^	0.12	−0.40^***^	0.01
Other-self divergence	−0.40^***^	0.02	−0.30^***^	0.03	−0.39^***^	0.01
Ambition	−0.01	−0.08	−0.04	−0.04	−0.02	−0.07
Need for sympathy	0.18^*^	−0.08	0.05	−0.13	0.14	−0.15

*N* = 465; Cohen’s *d* describes the self-other differences between the target and the mother or the friend; *p* < 0.05/0.01/0.001 corresponds to ^*^/^**^/^***^; Informant total as mean other-rating between mother and friend.

## 6 Discussion

The IP comprises feelings of fraudulence, dissociation from the authentic self, and the intention to conceal self-doubts. Consequently, a heightened IP expression leads to a biased self-representation and, therefore, to a less accurate external assessment. The supposedly negative association of the IP with the judge’s accuracy, indicated by less associated self- and other-rating, was the subject of this study.

Firstly, The IPP emerges as a suitable instrument for exploring the SOA, given its capacity to assess both the overall IP expression and its facets. In particular, we focused on the relationship between the profile agreement and the self-rated subscale expressions of Alienation (the feeling of not being able to be oneself) and Other-Self Divergence (the perceived inflated expectation by others). This study revealed two main findings:

1. The psychometric properties of the IPP informant version were suitable, with the examination of measurement invariance confirming scalar invariance, substantiating the validity of comparing the self and other versions of the IPP.
2. The profile agreement between the targets and informants was negatively related to the total impostor expression, Alienation and Other-Self Divergence, with effects disappearing when controlling for normativeness.

As a prerequisite, examining measurement invariance between the self and other IPP versions supported their comparative use psychometrically. Scalar invariance, indicating consistent factor structure, item loadings, and intercepts, supported the validity of the IPP other-version for future investigations and was the prerequisite for the profile-agreement analysis in this study (Mõttus et al., 2020).

The initial investigations of demographics revealed that IP inversely correlated with age, aligning with prior research findings (Mascarenhas et al., 2019). Notably, the subscale Ambition showed the strongest correlation. This result can be attributed to a more settled life stage, heightened realism in self-ambition, and, subsequently, greater tendencies toward frugality and self-compassion. An educational disparity between the child and the family and parents had been considered an important predictor on the literature (Clance, 1985). Therefore, in this study, we exploratory examined the association of the mothers’ and targets’ educational differences with the targets’ IP expression. The results indicate that the educational disparity was unrelated to the IP, weakening the assumption of family socioeconomic differences as its origin, also in line with the results by Sonnak and Towell (2001). Examining the relation between the IP and gender indicated, similar to previous studies, no gender difference in the overall IP expression, but the differences became apparent at the subscale level. Women show a higher expression in Competence Doubt and Alienation. In contrast, men show a higher expression in Working Style (Procrastination) and Ambition. However, in previous studies, no gender differences were found in the subscales Working Style and Alienation (Ibrahim et al., 2021). These differences could result from demographic characteristics, as the sample in this study differs significantly from the much younger student sample in Ibrahim et al. (2021).

Besides demographic relations, the main focus of this study was the association of the IP and the judge’s accuracy, operationalized by the profile-agreement analysis. Comparing the profile agreement between the two informants showed that friends demonstrated a slightly lower overall agreement than mothers. However, while the difference did not attain statistical significance, a bigger sample in future studies could support the acquaintanceship effect, as family members tend to exhibit higher profile agreement in self-other ratings compared to friends (Connelly and Ones, 2010). A closer relationship with the target facilitates a richer and more comprehensive understanding of the individual, enabling more precise assessments (Funder and Colvin, 1988). Especially regarding the hardly assessable IP, with its relation to impression management and attention to social comparison (Parkman, 2016), the relationship quality and number of observations in different scenarios is even more critical. In addition, the attributes of impostors are less external and behavioral than internal, characterized by self-related cognitions, attitudes, and feelings (Tigranyan et al., 2021). Those affected, therefore, show characteristics that reduce behavioral coherence, continuity, and open self-disclosure, resulting in a more challenging external assessment according to the Realistic Accuracy Model (RAM; Funder, 1995).

Interestingly, the aggregation of both other-ratings increases the SOA, attains greater accuracy and can be regarded as a comprehensive overview of others’ perceptions (Roth and Altmann, 2021). We also found a negative correlation between both informant’s distinctive agreements. This finding indicates that characteristics where the target and informant distinctly agree on differ. So, distinct features are assessed from various angles by the mother and friends, incrementally complementing each other. The low normative and distinctive inter-rater agreement between the external assessment of mother and friends also supports the complementary perspective assumption regarding both judges. Fiedler et al. (2004) also emphasize that low SOA does not inherently imply low validity. Therefore, self- and peer reports can complement each other by adding information (Furr et al., 2007).

Examining the relation between profile agreement and the target’s impostor expression revealed the assumed difference between internal and external impostor characteristics. Accordingly, there were no significant negative correlations between the SOA and Working Style, Ambition, and Need for Sympathy, as those traits are better observable. A higher expression in those subscales did not worsen the judge’s accuracy, aligning with the statement of Funder (1995) that trait visibility is associated with greater SOA. Moreover, due to their less intimate nature and more favorable connotations, these traits might prompt both the target and informant to provide a more accurate assessment. The Competence Doubt scale exhibited a negative relationship with the SOA in mothers, possibly because the mothers do less often experience their child during impostor tendency-evoking situations. In addition, mothers might tend to maintain a positively biased perception of their children and, therefore, underestimate their children’s competence doubts.

In contrast, among friends, Competence Doubt showed a positive correlation with the distinctive agreement rather than overall agreement. This finding could be explained by the tendency to self-report self-doubts to good friends, indicated by a more precise distinct other-rating, whereby the overall agreement did not correlate with Competence Doubt. The missing relation could be attributed to a dilution of distinctive ratings from good friends (more intimate self-revealing information) through mediocre friends answering more stereotypically.

The Alienation scale demonstrated a consistent negative correlation with the SOA for both friends and mothers, suggesting a less felt authentic behavior in social contexts contributes to a less accurate assessment by others. Similarly, the Other-Self Divergence scale displayed a negative relationship with the SOA, as the feeling of being overrated by others implies a high divergence between the targets and judges’ assessment inherently. These findings collectively emphasize that IP-specific characteristics like felt inauthenticity (Alienation) and the perception of being overestimated (Other-Self Divergence) are associated with lower profile agreement. In conclusion, individuals higher in IP, especially in the subscales Alienation and Other-Self Divergence, are less accurately assessed by even close others.

## 7 Limitations and implications

Indeed, our study has several limitations and opportunities for further improvement that deserve discussion. Foremost, it is crucial to acknowledge the presence of biases inherent to a construct like the IP. Given that impostors tend to hold distorted self-perceptions, underestimating themselves (Thompson et al., 1998), and displaying a polished self-presentation (Topping and Kimmel, 1985), an accurate other-assessment becomes inherently challenging (Letzring and Funder, 2018). Including a behavior-based external criterion could serve as a triangulation point of self- and other perceptions. Furthermore, surveying other judges in future studies, such as work colleagues, bosses, and teachers, would be insightful to generate further incremental information. In particular, work colleagues could exhibit a lower positivity bias. Here, it would be informative to examine whether long-term colleagues (knowing) without having a friendship (liking) attain a higher distinctive agreement since the informant’s attitude and desirability toward the target affects the assessment (Leising et al., 2015). Additionally, our survey did not determine the friendship intensity, quality, or shared interests and activities (e.g., hobbies, studying, work), which would be a profitable variable for future studies to contribute to the acquaintanceship effect (Brauer et al., 2023). The examination of relationship quality is essential, particularly when mothers are the judges, given the negative association between relationship quality and the IP (Garwick et al., 2011). This consideration is crucial due to its potential biasing influence when assessing the self-other agreement. Further, it would be necessary for future studies to survey fathers as judges, as previous results show that an overprotective father is positively related to IP expression (Want and Kleitman, 2006).

The psychometric properties of the IPP’s self- and other-versions are acceptable, although the Need for Sympathy scale showed low reliability. In addition, the sample consists of exclusively German participants. In order to make the results more representative, future SOA studies could encompass other cultural backgrounds. A prerequisite here would also be the validation of the other-versions of the IPP in English, which could be a goal of future studies.

With this study, we hope to increase interest in the self-other perspective of the IP, recognizing the potential value of integrating multiple viewpoints to enrich the comprehension of this construct. Implementing the informant perspective could support numerous research findings regarding the IP. For example, using the informant version, future research could further validate the typology of *strategic* and *true* impostors (Leonhardt et al., 2017). The deliberate self-presentation of strategic impostors could lead to a lower agreement, as they indicate self-doubt and anxiety but feel less dysthymia and agitation than true impostors. Further the investigation of the IP exhibition longitudinally from an informant perspective to examine influencing factors such as career changes, partnerships, or psychological interventions could lead to a better understanding of the constructs predictors. We also aspire to generate practical value with the validated IPP informant version, e.g., in psychological counseling and coaching. An IPP 360°-feedback could serve as a diagnostical ground, tailoring targeted interventions and monitoring progress. The comparison of the IPP self- and other-ratings could stimulate new diagnostic approaches, enrich the client’s self-knowledge, and improve the counselor’s therapeutic model.

The IP, characterized by a negatively distorted self-perception (Competence Doubt) and a biased external perception (Other-Self Divergence; Alienation), requires multiple perspectives to control for different biases. Therefore, a multi-rater approach facilitates understanding the construct’s emergence, change, and inherent mechanisms. Consequently, we encourage future studies to look at the metaphorical mask not only from the inside but also from the outside. Also, in a consultative context, the informant’s perspective from various reference persons could assist in understanding the dynamic of IP-specific features and support those affected to gain control and remove the mask in the long run.

## Data availability statement

The datasets presented in this study can be found in online repositories. The names of the repository/repositories and accession number(s) can be found at: https://www.osf.io/yh5g7.

## Ethics statement

The studies involving humans were approved by Ethics Committee of the Helmut-Schmidt-University; PH (chairman); ethikkommission.geiso@hsu-hh.de. The studies were conducted in accordance with the local legislation and institutional requirements. The participants provided their written informed consent to participate in this study.

## Author contributions

FI: Conceptualization, Data curation, Formal analysis, Investigation, Methodology, Supervision, Visualization, Writing – original draft, Writing – review & editing. EB: Conceptualization, Data curation, Investigation, Methodology, Project administration, Writing – review & editing. TM: Data curation, Project administration, Writing – review & editing. PH: Project administration, Supervision, Writing – review & editing.

## References

[ref1] Brauer K. Sendatzki R. Proyer R. T. (2023). Exploring the acquaintanceship effect for the accuracy of judgments of traits and profiles of adult playfulness. J. Pers. 1–20. doi: 10.1111/jopy.12839, PMID: 37041675

[ref2] Brauer K. Wolf A. (2016). Validation of the German-language Clance impostor phenomenon scale (GCIPS). Personal. Individ. Differ. 102, 153–158. doi: 10.1016/j.paid.2016.06.071

[ref1001] Bravata D. M. Watts S. A. Keefer A. L. Madhusudhan D. K. Taylor K. T. Clark D. M. (2020). Prevalence, predictors, and treatment of impostor syndrome: a systematic review. J. Gen. Intern. Med. 35, 1252–1275. doi: 10.1007/s11606-019-05364-1 31848865 PMC7174434

[ref9001] Brennan-Wydra E. Chung H. W. Angoff N. ChenFeng J. Phillips A. Schreiber J. (2021). Maladaptive Pectionism, impostor phenomenon, and suicidal ideation among medical students. Acad. Psychiatry. 45, 708–715. doi: 10.1007/s40596-021-01503-1 34350548

[ref3] Chen F. F. (2007). Sensitivity of goodness of fit indexes to lack of measurement invariance. Struct. Equ. Model. Multidiscip. J. 14, 464–504. doi: 10.1080/10705510701301834

[ref4] Clance P. R. (1985). The impostor phenomenon: Overcoming the fear that haunts your success. Atlanta: Peachtree Publishers (hardback).

[ref9002] Clance P. R. Imes S. A. (1978). The imposter phenomenon in high achieving women: dynamics and therapeutic intervention. Psychotherapy: Psychol. Psychother: Theory Res. Pract. 15, 241–247. doi: 10.1037/h0086006

[ref5] Connelly B. S. Ones D. S. (2010). An other perspective on personality: Meta-analytic integration of observers’ accuracy and predictive validity. Psychol. Bull. 136, 1092–1122. doi: 10.1037/a0021212, PMID: 21038940

[ref6] Ferrari J. R. Thompson T. (2006). Impostor fears: links with self-presentational concerns and self-handicapping behaviours. Personal. Individ. Differ. 40, 341–352. doi: 10.1016/j.paid.2005.07.012

[ref7] Fiedler E. R. Oltmanns T. F. Turkheimer E. (2004). Traits associated with personality disorders and adjustment to military life: predictive validity of self and peer reports. Mil. Med. 169, 207–211. doi: 10.7205/MILMED.169.3.207, PMID: 15080240 PMC4380139

[ref8] Funder D. C. (1995). On the accuracy of personality judgment: a realistic approach. Psychol. Rev. 102, 652–670. doi: 10.1037/0033-295X.102.4.652, PMID: 7480467

[ref9] Funder D. C. Colvin C. R. (1988). Friends and strangers: acquaintanceship, agreement, and the accuracy of personality judgment. J. Pers. Soc. Psychol. 55, 149–158. doi: 10.1037/0022-3514.55.1.149, PMID: 3418488

[ref10] Furr R. M. (2008). A framework for profile similarity: integrating similarity, normativeness, and distinctiveness. J. Pers. 76, 1267–1316. doi: 10.1111/j.1467-6494.2008.00521.x, PMID: 18705644

[ref11] Furr R. M. Dougherty D. M. Marsh D. M. Mathias C. W. (2007). Personality judgment and personality pathology: self-other agreement in adolescents with conduct disorder. J. Pers. 75, 629–662. doi: 10.1111/j.1467-6494.2007.00451.x, PMID: 17489894

[ref9003] Garwick M. R. Ford A. C. Hughes J. L. (2011). Impostor phenomenon and females’ self-esteem, GPA, and relationship with mother. J. Undergrad. Res. Int. j. human sci. 10.

[ref12] Ibrahim F. Göddertz D. Herzberg P. Y. (2022c). An experimental study of the non-self-serving attributional bias within the impostor phenomenon and its relation to the fixed mindset. Curr. Psychol. 42, 26440–26449. doi: 10.1007/s12144-022-03486-0

[ref13] Ibrahim F. Münscher J.-C. Herzberg P. Y. (2021). Examining the impostor-profile—is there a general impostor characteristic? Front. Psychol. 12:720072. doi: 10.3389/fpsyg.2021.720072, PMID: 34566801 PMC8458651

[ref14] Ibrahim F. Münscher J.-C. Herzberg P. Y. (2022a). The facets of an impostor – development and validation of the impostor-profile (IPP31) for measuring impostor phenomenon. Curr. Psychol. 41, 3916–3927. doi: 10.1007/s12144-020-00895-x

[ref15] Ibrahim F. Münscher J.-C. Herzberg P. Y. (2022b). The validation of the English impostor-profile 30 and the exploratory formulation of the learned helplessness model of the impostor phenomenon. Acta Psychol. (Amst) 226:103589. doi: 10.1016/j.actpsy.2022.103589, PMID: 35427931

[ref9004] Jöstl G. Bergsmann E. Lüftenegger M. Schober B. Spiel C. (2012). When will they Blow my cover? Zeitschrift Für Psychologie, 220, 109–120. doi: 10.1027/2151-2604/a000102

[ref16] Kenrick D. T. Funder D. C. (1988). Profiting from controversy: lessons from the person-situation debate. Am. Psychol. 43, 23–34. doi: 10.1037/0003-066X.43.1.23, PMID: 3279875

[ref17] Klinkhammer M. Saul-Soprun G. (2009). The impostor syndrome in the academic and scientific area. Organ. Superv. Coach. 16, 165–182. doi: 10.1007/s11613-009-0119-7

[ref18] Kolligian J. Sternberg R. J. (1991). Perceived fraudulence in young adults: is there an’imposter syndrome’? J. Pers. Assess. 56, 308–326. doi: 10.1207/s15327752jpa5602_10, PMID: 2056424

[ref19] Leising D. Scherbaum S. Locke K. D. Zimmermann J. (2015). A model of “substance” and “evaluation” in person judgments. J. Res. Pers. 57, 61–71. doi: 10.1016/j.jrp.2015.04.002

[ref20] Leonhardt M. Bechtoldt M. N. Rohrmann S. (2017). All impostors Aren't alike—differentiating the impostor phenomenon. Front. Psychol. 8:1505. doi: 10.3389/fpsyg.2017.01505, PMID: 28936188 PMC5594221

[ref1002] Letzring T. D. Funder D. C. (2018). Interpersonal accuracy in trait judgments. The SAGE handbook of personality and individual differences. 3, 253–282.

[ref21] Mascarenhas V. R. D’Souza D. Bicholkar A. (2019). Prevalence of impostor phenomenon and its association with self-esteem among medical interns in Goa, India. Int J Commun Med Public Health 6, 355–359. doi: 10.18203/2394-6040.ijcmph20185272

[ref22] Mõttus R. Wood D. Condon D. M. Back M. D. Baumert A. Costantini G.. (2020). Descriptive, predictive and explanatory personality research: different goals, different approaches, but a shared need to move beyond the big few traits. Eur. J. Pers. 34, 1175–1201. doi: 10.1002/per.2311

[ref23] Noskeau R. Santos A. Wang W. (2021). Connecting the dots between mindset and impostor phenomenon, via fear of failure and goal orientation, in working adults. Front. Psychol. 12:588438. doi: 10.3389/fpsyg.2021.588438 34867567 PMC8636168

[ref24] Parkman A. (2016). The imposter phenomenon in higher education: incidence and impact. J High Educ Theory Pract 16, 51–60.

[ref9005] R Core Team (2017). R: A language and environment for statistical computing. R Foundation for Statistical Computing, Vienna, Austria. Available at: https://www.R-project.org/.

[ref25] Reise S. P. Moore T. M. Haviland M. G. (2010). Bifactor models and rotations: exploring the extent to which multidimensional data yield univocal scale scores. J. Pers. Assess. 92, 544–559. doi: 10.1080/00223891.2010.496477, PMID: 20954056 PMC2981404

[ref27] Rosseel Y. Oberski D. Byrnes J. Vanbrabant L. Savalei V. Merkle E.. (2017). Package ‘lavaan.’ Available at: https://cran.r-project.org/web/packages/lavaan/lavaan.pdf (Accessed 17 June 2017).

[ref28] Roth M. Altmann T. (2021). The self-other agreement of multiple informants on empathy measures and its relation to empathic accuracy. Personal. Individ. Differ. 171:110499. doi: 10.1016/j.paid.2020.110499

[ref29] Seto E. Schlegel R. J. (2018). Becoming your true self: perceptions of authenticity across the lifespan. Self Identity 17, 310–326. doi: 10.1080/15298868.2017.1322530

[ref30] Sonnak C. Towell T. (2001). The impostor phenomenon in British university students: relationships between self-esteem, mental health, parental rearing style and socioeconomic status. Personal. Individ. Differ. 31, 863–874. doi: 10.1016/S0191-8869(00)00184-7

[ref31] Thompson T. Davis H. Davidson J. (1998). Attributional and affective responses of impostors to academic success and failure outcomes. Personal. Individ. Differ. 25, 381–396. doi: 10.1016/S0191-8869(98)00065-8

[ref32] Tigranyan S. Byington D. R. Liupakorn D. Hicks A. Lombardi S. Mathis M.. (2021). Factors related to the impostor phenomenon in psychology doctoral students. Train Educ Profess Psychol 15, 298–305. doi: 10.1037/tep0000321

[ref33] Topping M. E. Kimmel E. B. (1985). The imposter phenomenon: feeling phony. Acad. Psychol. Bull. 7, 213–226.

[ref34] Vazire S. Carlson E. N. (2010). Self-knowledge of personality: do people know themselves?: self-knowledge of personality. Soc. Personal. Psychol. Compass 4, 605–620. doi: 10.1111/j.1751-9004.2010.00280.x

[ref35] Vergauwe J. Wille B. Feys M. De Fruyt F. Anseel F. (2015). Fear of being exposed: the trait-relatedness of the impostor phenomenon and its relevance in the work context. J. Bus. Psychol. 30, 565–581. doi: 10.1007/s10869-014-9382-5

[ref36] Want J. Kleitman S. (2006). Imposter phenomenon and self-handicapping: links with parenting styles and self-confidence. Personal. Individ. Differ. 40, 961–971. doi: 10.1016/j.paid.2005.10.005

[ref37] Yaffe Y. (2021). Students’ recollections of parenting styles and impostor phenomenon: the mediating role of social anxiety. Personal. Individ. Differ. 172:110598. doi: 10.1016/j.paid.2020.110598

