# Supplementary material for: The impostor phenomenon in the eye of knowledgeable others: the association of the impostor phenomenon with the judge’s accuracy
Source: Front Psychol. 2023 Dec 22;14:1290686. doi: 10.3389/fpsyg.2023.1290686 (PMC10766844; doi:10.3389/fpsyg.2023.1290686)
Supplement: Supplementary file 1 [file Data_Sheet_1.PDF]

Supplementary Material

Table A.1: *Sample Characteristics of the Self- and Other-Rating in Total and solely for Targets, Friends and Mothers*

		Target	Mother	Friend	Total self	Total other
		155	155	155	465	310
Age	Age	25.79 (*SD* = 7.77)	55.36 (*SD* = 7.59)	25.90 (*SD* = 7.52)	35.68 (*SD* = 15.86)	40.62 (*SD* = 16.56)
	Gender (♀)	90 (58)	155 (100)	78 (50)	323 (69)	233 (75)
Education level	No educational attainment	1 (1)	0	1 (1)	2 (<1)	1 (<1)
	Certificate of secondary Education	0	13 (8)	3 (2)	16 (3)	16 (5)
	Secondary school level I certificate	1 (1)	30 (19)	14 (9)	45 (10)	44 (14)
	School leaving examination	68 (44)	23 (15)	64 (41)	155 (33)	87 (28)
	Vocational education	20 (13)	50 (32)	29 (19)	99 (21)	79 (25)
	Bachelor degree	53 (34)	2 (1)	32 (21)	87(19)	34 (11)
	Master's degree or higher	12 (8)	37 (24)	12 (8)	61 (13)	49 (16)
Occupation	Unemployed	0	29 (19)	5 (3)	34 (7)	34 (11)
	Domestic activities	3 (2)	12 (8)	4 (3)	19 (4)	16 (5)
	Student	89 (57)	0	73 (47)	162 (35)	73 (24)
	employed < 30h/ week	9 (6)	44 (28)	4 (3)	57 (12)	48 (15)
	employed > 30h/ week	54 (35)	62 (40)	68 (44)	184 (40)	130 (42)
	Pensioner	0	8 (5)	1 (1)	9 (2)	9 (3)

Note. The persentages have been rounded to whole numbers.

Table A.2: Measurement Invariance between the Impostor-Profile Target- and Informant-Version

Models		χ^2 (*df*)	CFI	RMSEA	SRMR
Models					
Model 1:	Configural invariance	1528.345 (750)	.932	.055	.053
Model 2:	Metric invariance	1669.803 (803)	.924	.056	.066
Model 3:	Scalar invariance	1942.018 (833)	.903	.062	.081
Model comparisons		$\Delta\chi 2$ (Δdf)	ΔCFI	ΔRMSEA	ΔSRMR
M2 - M1		141.46 (53)	-.008	.001	.013
M3 - M2		272.22 (30)	-.021	.006	.015

Note. The measurement invariance was calculated with the bifactor model (one bifactor and six group factors); CFI = Comparative Fit Index; RMSEA = Root Mean Square Error of Approximation; SRMR = Standardized Root Mean Squared. Residual.
